# Long-term efficacy and safety of biologics in rheumatoid arthritis

**DOI:** 10.1186/ar3719

**Published:** 2012-03-08

**Authors:** Carlomaurizio Montecucco

**Affiliations:** 1 Division of Rheumatology, University of Pavia, IRCCS Policlinico S. Matteo, Pavia, Italy

## 

Biologic agents have been used for more that twelve years in treating rheumatoid arthritis (RA) and other rheumatic diseases. Long-term analysis for both efficacy/effectiveness and safety is now feasible for the first anti-TNF agents available, namely infliximab, etanercept and adalimumab. Open-label extension studies have shown that clinical improvement is maintained up to10 years. However, these studies include only the patients who have sustained efficacy without the development of significant adverse events that would cause the patient to exit the study prior to closure. Therefore, as with any completer analysis of efficacy, the results reported are generally positive. Furthermore, clinical trial patients may not be representative of the entire patient population since in the clinic setting the indications for treatment with biologic agents are not identical to the inclusion criteria for trials. Only a minority of the patients in the clinical setting would have been eligible for the major trials; ineligible patients have lower baseline disease activity, more comorbidity, lower functional status and lower response rates. Despite these data, long term evaluation of effectiveness and safety in observational studies and registers has shown that TNF inhibitors are still a good choice in treatment of RA and may be also associated with a reduced risk of cardiovascular events in patients with RA.

